# The Tri-State Medical Society of Alabama, Georgia, and Tennessee

**Published:** 1894-09

**Authors:** 


### Editorial.

### THE TRI-STATE MEDICAL SOCIETY OF ALABAMA, GEORGIA, AND TENNESSEE.

The sixth annual meeting of the above Society will be held in Atlanta, Ga., on October 9th, 10th and 11th. This is the first time that this Association has met outside of Chattanooga, and there is expected quite a large number of members, as wel! as visitors from different sections.

It is proposed that the name and scope of the Association be materially changed, so as to widen its field of usefulness by including all or a major part of the South. The name of "Southeastern, or Southern Medical Society" will probably be adopted. Every Southern physician is urged to attend this meeting, which will be the largest ever held in the South. The influence of all the physicians of the South can hardly be estimated, when associated together in such an organization as this is to be made, and we earnestly invite the influence and participation of every one of our readers in this matter.

As you see from the partial list below, while we have a lot of valuable papers from prominent Southern physicians, there will also be read papers by Jno. A. Wyeth, M.D., and W. Gill Wylie of New York, Dr. Jos. Price of Philadelphia, and others who have promised their presence.

The city of Atlanta will give a hospitable reception to all visitors, and a good time may be expected by all. Reduced rates will be given on all railroads, on the certificate plan.

The following are the present officers:

President—Dr. J. B. S. Holmes, Atlanta.

Vice-Presidents—Dr. Jas. A. Goggans, Alexander City, Ala.; Dr. Dan H. Howell, Atlanta, Ga.; Dr. T. Hilliard Wood, Nashville, Tenn.

Secretary—Dr. Frank Trester Smith, Chattanooga, Tenn.

Treasurer—Dr. W. C. Townes, Chattanooga, Tenn.

Recorder—Dr. W. L. Gahagan, Chattanooga, Tenn.

Partial list of papers:

The Reponsibility of a Class of Criminals from a Medico-Legal Point of View, J. C. LeGrand, Anniston, Ala.; Treatment of Stricture of the Urethra by Electrolysis, P. L. Brouillette, Huntsville, Ala.; Urethral Surgery Ten Years ago and To-day, T. C. V. Barkley, Chattanooga; Reflex Neurosis in the Male, Andrew Boyd, Scottsboro, Ala.; The Pathological Import of Albumen in the Urine, E. B. Ward, Selma, Ala.; How to do Abdominal Section without Fuss, Feathers or Foolishness, and with Immunity from Sepsis, Joseph Price, Philadelphia; Puerperal Septicemia, with Cases Illustrating the Several Varieties, J. R. Rathmell, Chattanooga; Essentials of Obstetric Nursing, R. R. Kime, Atlanta; Pernicious (or Inveterate) Vomiting of Pregnancy—A Plea for the Mother; Based on Cases in Actual Practice, E. A. Cobleigh, Chattanooga; The Induction of Labor to Prevent Blindness, Frank Trester Smith, Chattanooga; Slaughter of the Innocents, E. van Goidtsnoven, Atlanta; Prognosis and Treatment of Placenta Previa, Richard Douglas, Nashville; Uterine Cancer, Geo. R. West, Chattanooga; Treatment of Uterine Fibroids, W. Gill Wylie, New York; A Report of Some Rare Surgical Lesions Connected with the Liver, John A. Wyeth, New York; The Treatment of Stone in the Kidney, W. E. B. Davis, Birmingham, Ala.; Tuberculosis of the Kidney and Bladder, H. Berlin, Chattanooga; Some Causes Leading to Invalidism in Women, J. B. S. Holmes, Atlanta; Amputation of the Mamma for Carcinoma and Treatment of the Axilla, B. W. Bizzell, Atlanta; Excision of Malignant Tumors of the Breast, Willis F. Westmoreland, Atlanta; Some Remarks upon Brain Surgery, with Report of Cases, Paul F. Eve, Nashville; The Surgical Treatment of Empyema, J. A. Goggans, Alexander City, Ala.; Appendicitis; Its Surgical Treatment, with Report of Cases, R. J. Trippe, Chattanooga; The Treatment of Injuries and Inflammation of the Joints, Wm. L. Nolen, Chattanooga; Burns and the Treatment Thereof, T. Ellis Drewry, Griffin; Is there Danger of not Getting Good Union after Tenotomy? C. W. Barrier, Columbus; Hygienic Treatment of Syphilis, T. M. Baird,

Hot Springs, Ark.; Electro-Therapeutics, J. P. Stewart, Chattanooga; Headaches; Their Etiology and Treatment, R. P. Johnson, Chattanooga; Migraine; Its Etiology and Treatment, Hugh Hagan, Atlanta; Tuberculosis of the Nasal Bone, B. F. Travis, Chattanooga; Paresis and Paralysis of the External Rectus of the Eye, with Report of Two Cases, Dunbar Roy, Atlanta; The Use of Hydrastis Canadensis in Diseases of Mucous Membranes, P. R. Cortelyou, Marietta; Combination of Carbolic Acid and Camphor as an Antiseptic and Local Anesthetic, William Perrin Nicolson, Atlanta; Some Practical Points in the Treatment of Typhoid Fever, James B. Baird, Atlanta; Treatment of Pneumonia in Children, Frank S. Parsons, Philadelphia; Adenoids and their Sequelæ, Arthur G. Hobbs, Atlanta; The Treatment of Smallpox, C. H. Holland, Chattanooga; An Outline of the History of Medicine and Surgery in Georgia, L. B. Grandy, Atlanta; Hypertrophic Rhinitis; Some of the Reflexes, George Brown, Atlanta; Some Surgical Results Described and Illustrated by T. H. Huzza, Atlanta, Ga.; Mixed Infection, M. B. Hutchins, Atlanta, Ga.; Some Points in Rectal Surgery, J. M. Mathews, Louisville, Ky.; Reform in the Treatment of the Neurotic and the Insane, Viewed from the Gynecological Standpoint, Charles A. L. Reed, Cincinnati, O.

